# Sex-Based Differences in Lifestyle Behaviours, Self-Esteem, and Academic Performance: A Structural Equation Model in High-Socioeconomic-Status School-Aged Youth from Southern Spain

**DOI:** 10.3390/children12111459

**Published:** 2025-10-27

**Authors:** Gracia Cristina Villodres, Juan-José Pérez-Díaz, José-Antonio Salas-Montoro, José Joaquín Muros

**Affiliations:** 1 Department of Didactics of Body Expression, Faculty of Education, University of Granada, 18071 Granada, Spain; gcvillodres@ugr.es; 2 Department of Physical Education and Sports, Faculty of Sports Sciences, University of Granada, 18071 Granada, Spain; jjpd@ugr.es (J.-J.P.-D.); salasmontoro@ugr.es (J.-A.S.-M.)

**Keywords:** screen time, sleep time, physical activity, Mediterranean diet, body mass index, self-esteem, academic performance, school-aged youth, structural equation model

## Abstract

**Highlights:**

**What are the main findings?**

- Sleep time (SLT) was positively associated with Mediterranean diet (MD) adherence and negatively associated with body mass index (BMI). Both physical activity engagement (PA) and MD adherence were positively related to self-esteem (SE), while MD adherence and SE were positively associated with academic performance (AP). These associations remained consistent regardless of socioeconomic status (SES) groups.
- Screen time (ST) emerged as a particularly influential factor among girls, showing negative associations with both PA and MD adherence. These patterns were not observed in boys.
- While a positive association between PA and SE was evident in both sexes, this relationship was stronger in boys.
- In the girls’ model, both MD adherence and SE were positively associated with AP, although this association was not found among boys.

**What are the implications of the main findings?**

- Interventions should go beyond merely promoting healthy behaviours and include psychological factors such as SE.
- Sex-specific tailoring of interventions is essential to address the distinct needs and patterns observed in boys and girls.
- Early intervention is critical to prevent age-related declines in PA and AP.

**Abstract:**

Objectives: The present study aimed to examine the relationships between screen time (ST), sleep time (SLT), physical activity engagement (PA), Mediterranean diet (MD) adherence, body mass index (BMI), self-esteem (SE) and academic performance (AP) in high-socioeconomic-status (SES) school-aged youth in southern Spain. Methods: A descriptive, comparative, non-experimental and cross-sectional research study was conducted with a total sample of 217 Spanish students (13.88 ± 1.32). Structural equation modelling (SEM) was employed to analyse relationships between study variables as a function of sex. Results: SLT was positively associated with MD adherence and negatively related with BMI. Both PA and MD adherence were positively linked to SE, whilst MD adherence and SE were also positively related to AP. Regarding sex differences, ST was a stronger determinant among girls, showing negative associations with PA (β = −0.378; *p* < 0.001) and MD adherence (β = −0.315; *p* < 0.001), with this pattern not being observed in boys. PA was positively associated with SE in both sexes, but more strongly in boys (β = 0.332; *p* < 0.001) than in girls (β = 0.190; *p* = 0.034). In girls, both MD adherence (β = 0.295; *p* < 0.001) and SE (β = 0.224; *p* = 0.008) were positively associated with AP, with these associations not being found in boys. Conclusions: The findings underscore the complex interplay between lifestyle behaviours, psychosocial factors, and AP in school-aged youth. Regardless of SES, interventions should focus on reducing ST, promoting PA and MD adherence, and enhancing SE whilst considering sex-specific patterns.

## 1. Introduction

The study of healthy habits, such as physical activity engagement (PA), healthy sleep habits and diet in school-aged youth, has been of great interest in recent decades. During adolescence, substantial biological, cognitive and psychosocial changes influence lifestyle behaviours and academic outcomes. Increased autonomy, digital media exposure, and concern with body image and peer relations can alter sleep patterns, self-esteem (SE), diet and PA engagement, ultimately shaping both health and educational trajectories [1].

Studies on Spanish school-aged youth have concluded that engaging in healthy habits, such as higher levels of PA, Mediterranean diet (MD) adherence, reduced screen time (ST) and sufficient sleep time (SLT), leads to improvements in both physical and mental health, as well as academic performance (AP) [2,3]. These findings are consistent with international research, which has also examined the associations between lifestyle behaviours and educational outcomes. Global evidence from diverse contexts indicates that factors such as PA engagement, sleep and diet quality are linked to academic and cognitive performance among youth [1,4,5]. Nevertheless, results remain mixed, as some studies have reported small or non-significant effects once sociodemographic and psychological factors are considered [6].

In this regard, the World Health Organization (WHO) recommends that school-aged youth engage in at least 60 min of moderate-to-vigorous physical activity (MVPA) per day [7]. Scientific evidence suggests that higher amounts and intensities of PA are associated with greater cardiorespiratory fitness, muscular strength, bone health and cardiometabolic health [8]. Moreover, PA has been associated with improved cognitive functions including attention, memory and concentration. All of these are essential for academic success [9]. During the challenging developmental school stage, PA also plays a crucial role in promoting mental health by fostering higher levels of SE [10].

Another key health-related habit that has been studied in schoolchildren is adherence to a healthy diet, particularly the MD in the Spanish population [11]. This traditional dietary pattern is characterized by a high consumption of vegetables, fruits, whole grains, nuts and seeds, as well as daily intake of olive oil, moderate consumption of cheese, yogurt, eggs and fish, and limited intake of red meat [12]. A recent systematic review suggests that MD adherence may act as a protective factor for mental health, potentially reducing the risk of conditions such as attention-deficit/hyperactivity disorder (ADHD), depression and anxiety [13]. Recent studies have also reported a positive association between MD adherence and SE among Spanish youth [14]. Furthermore, MD adherence has been positively associated with AP in another similar population [15].

In addition, it is important to recognise that health-promoting behaviours, such as higher consumption of fruits and vegetables, regular intake of dairy products, consistently eating a healthy breakfast, following a more nutritious diet, and maintaining a physically active lifestyle, are more commonly observed among school-aged youth from families with a higher socioeconomic status (SES) [8].

Despite the well-documented benefits of these behaviours, WHO reports that approximately 80% of school-aged youth worldwide do not meet recommended PA levels. In the Spanish context, the Gasol Foundation [16], through the ‘PASOS Study’, highlighted a concerning decline in PA among eight to 16 year olds, along with a more than 10 percent decrease in MD adherence in this population. Moreover, longitudinal monitoring between 2022 and 2025 showed an alarming annual increase of 590.6 h in ST, which is equivalent to 25 full days (24 h a day) [16]. Notably, only 34.9% of Spanish school-aged youth meet the established PA recommendations [16].

According to Romero-Roso et al. [17], Spanish school-aged youth who do not meet the recommended sleep duration of eight to 10 h per night tend to spend more time using video games, the Internet and mobile phones. It is also common for young people to use their phones immediately before bedtime, a habit that has been identified as detrimental to sleep quality [18]. This screen-induced sleep deprivation has been associated with difficulties waking up, increased daytime sleepiness, irritability and feelings of sadness [19].

Moreover, the bidirectional relationship between excessive ST and sleep deprivation has been associated with increased sedentary behaviour and a greater risk of overweight or obesity among Spanish school-aged youth [20]. As previously noted, the combination of prolonged screen use and insufficient sleep contributes not only to a more sedentary lifestyle but also to higher consumption of unhealthy foods and an increase in body mass index (BMI) beyond recommended levels [21].

Low PA engagement, mediated by body image perception, has been shown to negatively affect SE in young people [22]. Notably, Delgado-Floody et al. [23] found that school-aged youth with an unhealthy BMI reported significantly lower SE compared to their peers with a healthy BMI. In this context, a higher BMI has commonly been linked to lower body appreciation, reduced body respect and decreased resistance to media pressures among schoolchildren [24]. At school age, complex relationships often exist between BMI and body image, with a resultant influence on SE. This underscores the importance of early interventions aimed at preventing eating disorders and related psychological issues [25].

Recent evidence indicates that certain healthy habits, such as higher levels of PA, strong MD adherence and reduced ST, are positively associated with better AP in Spanish school-aged youth [2]. However, these lifestyle factors are not absolute determinants of AP. Mental health variables may also play a significant role. For instance, in youth populations, SE has been found to moderate the relationship between cognitive abilities and AP [26]. The previously mentioned alarming rates of physical inactivity, increased ST and low adherence to healthy dietary patterns may be negatively affecting AP among school-aged youth. In this regard, the PISA 2022 report shows a substantial decline in the performance of Spanish youth in terms of mathematics, reading and science [27].

Finally, sex differences should be considered when examining these interrelated variables. According to Franco-Arévalo et al. [28], males exhibit higher levels of PA than females in both primary and secondary education, with a marked increase in physical inactivity among females during the transition to secondary school. Nevertheless, Spanish female students tend to outperform their male peers in terms of AP [29].

Thus, the aim of the present study was to examine the relationships between ST, SLT, PA, MD adherence, BMI, SE and AP in school-aged youth with a high SES in southern Spain.

To achieve this aim, the study pursued the following objectives:

- Develop an explanatory model of the relationships between ST, SLT, PA, MD adherence, BMI, SE, and AP in school-aged youth.
- Analyse the associations among the variables included in the model according to sex.

### 1.1. Current Study

While previous research has consistently shown that healthy lifestyle behaviours, such as PA, MD adherence and adequate sleep, are associated with better psychological well-being and AP, few studies have examined the complex interrelationships among these variables within a comprehensive explanatory model. Even fewer have considered the way in which these associations may differ according to sex, despite well-established differences in health behaviours and AP between males and females.

Furthermore, whilst having a high SES is typically associated with greater access to health-promoting resources, school-aged youth from affluent families are not exempt from facing challenges such as excessive ST, low SE, and other psychosocial difficulties. This is particularly relevant given that such issues are often attributed primarily to socioeconomic disadvantage. Thus, when these issues persist, even in high-SES populations, it becomes essential to explore the other factors that might be at play.

The present study addresses this gap by developing a structural equation model that integrates ST, SLT, PA, MD adherence, BMI and SE as predictors of AP in school-aged youth with a high SES. This approach aims to provide a more nuanced understanding of the way in which these variables interact, both directly and indirectly, and whether the strength or direction of these associations varies according to sex.

### 1.2. Theoretical Framework

The present study is grounded in self-determination theory [30], which emphasises the role of intrinsic motivation and the satisfaction of basic psychological needs (autonomy, competence and relatedness) in promoting adaptive behaviours and well-being. Within this framework, PA engagement and adherence to a healthy diet can be interpreted as self-determined behaviours that contribute to enhanced SE and, consequently, improved AP.

Additionally, the study is informed by Bronfenbrenner’s ecological systems theory [31], which posits that individual development results from interactions between personal characteristics and environmental contexts, including family, peers and school. This perspective supports the inclusion of lifestyle variables such as ST and SLT, which are strongly influenced by environmental and social factors, as exogenous determinants of health and academic outcomes.

Integrating these theoretical perspectives provides a coherent conceptual basis for the proposed structural equation model. Specifically, ST and SLT are conceptualised as exogenous variables that influence lifestyle behaviours (PA, MD and BMI). These, in turn, impact psychosocial factors such as SE, which ultimately relate to AP. The model, therefore, reflects both behavioural and psychological pathways through which lifestyle factors may influence educational outcomes.

## 2. Materials and Methods

### 2.1. Design and Participants

A descriptive, comparative, non-experimental, and cross-sectional research design was employed. The sample included 217 students from a mixed-funding school located in the city of Granada, Spain. Of these, 122 were girls (56.2%) and 95 were boys (43.8%), with a mean age of 13.88 years (SD = 1.32).

The selection of participants was carried out through non-probability, convenience sampling. Participation was voluntary, and informed consent was obtained in advance from the students’ parents or legal guardians. The study was approved by the school’s administration as well as the relevant parents’ associations.

In addition, SES was assessed using the FAS III instrument, and all participants were classified as having a high SES.

### 2.2. Instruments

#### 2.2.1. Physical Activity

Youth’s engagement in PA was measured using the Spanish-adapted version of the Physical Activity Questionnaire for Adolescents (PAQ-A) [32], which has been previously validated for use with participants aged 13 to 17 years. This self-report tool contains 10 items that assess moderate-to-vigorous PA levels during the seven days prior to completing the questionnaire. A global PA score is calculated by averaging the responses to the first nine items, which are rated along a five-point Likert scale. The final item serves as a control to identify any external factors that may have limited PA during the recall period. No participant marked this option.

#### 2.2.2. Mediterranean Diet Adherence

Dietary habits were evaluated using the updated version of the KIDMED questionnaire developed by Altavilla et al. [33]. This instrument consists of 16 dichotomous (yes/no) items that assess adherence to the Mediterranean dietary pattern in children and adolescents. Twelve items are positively framed, with ‘yes’ responses being scored as +1, while four items are negatively framed, with ‘yes’ responses being scored −1. ‘No’ responses receive a score of 0. Total scores range from −4 to 12. A higher score indicates greater MD adherence.

#### 2.2.3. Self-Esteem

SE levels were assessed using a validated Spanish version of the Rosenberg Self-Esteem Scale [34,35]. This scale consists of 10 items, with five being positively framed and five being negatively framed. Responses are rated from A to D and are scored accordingly (4 to 1 for positive items; 1 to 4 for negative ones). This structure is intended to mitigate response bias. Higher scores indicate greater self-esteem.

#### 2.2.4. Academic Performance

Students’ AP was assessed using their official school grades from the first term of the 2024–2025 academic year. The participating school provided the students’ marks in nine core subjects: Natural Sciences, Social Sciences, Spanish Language and Literature, Mathematics, English, Religion or Ethical Values, Artistic Education, Physical Education, and French. The final score was calculated as the average grade across the nine subjects.

Although grading criteria may vary slightly among teachers, all grades were derived from standardised curricular assessments conducted in line with national educational guidelines. These grades are, therefore, considered to be a valid and comparable indicator of students’ AP within this educational setting, as observed in longitudinal studies [36].

#### 2.2.5. Ad hoc Questionnaire

An ad hoc questionnaire was developed to assess screen time, sleep time, BMI, and sociodemographic data.

Firstly, to quantify sedentary behaviour related to digital media use, participants completed a custom-designed questionnaire that included items on the amount of time spent daily on screen-based leisure activities. These activities included watching TV, using mobile phones, playing video games, and working on computers. Students reported screen usage separately for weekdays and weekends. A composite score was then calculated by averaging the total daily hours across all seven days of the week.

Secondly, participants reported their usual bedtime and wake-up time during both weekdays and weekends. An average sleep schedule was then calculated by averaging these times to obtain a representative weekly sleep profile for each student.

In addition, participants reported their sex, date of birth, height and weight. BMI was then calculated from height and weight (BMI = weight [kg]/(height [m])^2^).

### 2.3. Procedure

Two distinct sets of informational materials were prepared to obtain informed consent. One was aimed at the directors of the educational institutions, whilst the other was intended for the parents or legal guardians of the participating school-aged young person. These materials provided detailed explanations of the study’s purpose, procedures, and requirements. Additionally, a research assistant was available to support and guide participants during the completion of questionnaires. Instructions on how to properly complete the questionnaires were given to all participants. All data collection and testing took place during school hours. Responses from a total of 67 participants were discarded from the 284 initially invited for the following reasons: (1) submission of informed consent signed by parents or legal guardians declining participation (*n* = 4), (2) failure to submit informed consent prior to the start of the evaluation (*n* = 9), (3) incomplete completion of the pre-post assessment questionnaires (*n* = 34), (4) refusal to provide academic grades (*n* = 20). The study received ethical clearance from the Ethics Committee of the University of Granada (4260/CEIH/2024) Data was gathered in November and December 2024.

### 2.4. Data Analysis

Data were analysed using IBM SPSS Statistics version 25.0. Sample distribution was assessed through the Kolmogorov–Smirnov test. As the results indicated a non-normal distribution, the Mann–Whitney U test was applied to compare the two independent groups. Partial correlations were computed while controlling for sex, with the level of statistical significance set at 0.05. Outcomes are displayed below in a heatmap.

**Figure 1:**
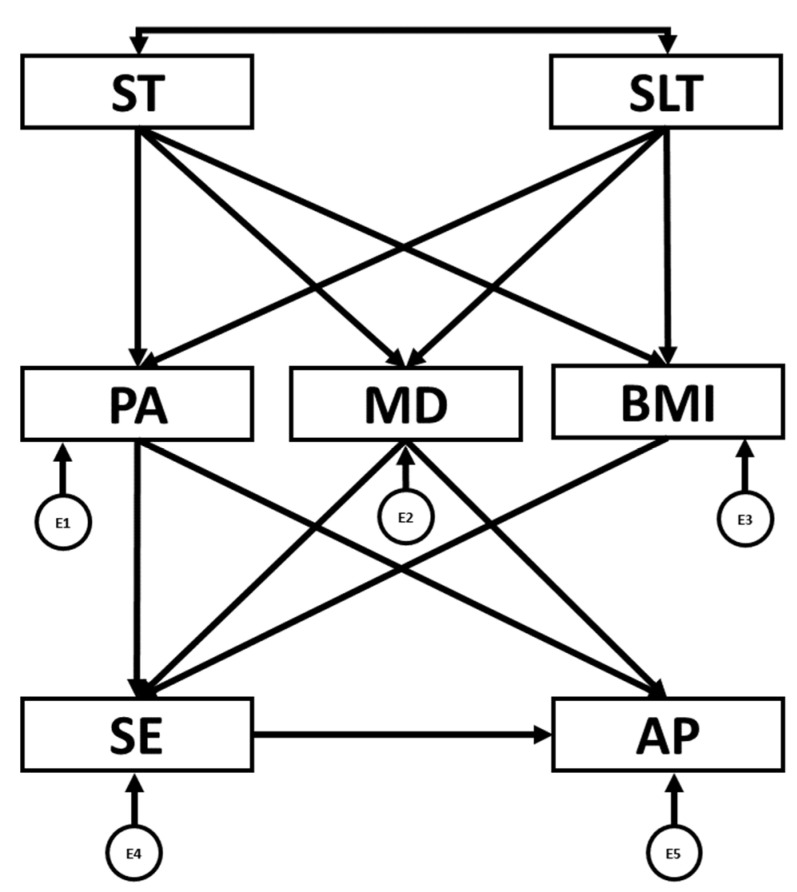
Structural equation model. PA: physical activity engagement; ST: screen time; SLT: sleep time; MD: Mediterranean diet; BMI: body mass index; SE: self-esteem; AP: academic performance. E1–E5: error terms. ←: directional relationships between variables; ↔: correlation between two variables.

Additionally, IBM AMOS^®^ version 24.0 software was employed to conduct structural equation modelling (SEM). This method enabled exploration of the relationships among the variables included in the theoretical model (Figure 1). The model comprised seven observed variables, including five endogenous (PA, MD adherence, BMI, SE and AP) and two exogenous variables (ST and SLT). Endogenous variables were associated with error terms, represented by circles, whereas exogenous variables were depicted with two-headed arrows, as they do not include error terms. SEM was used to examine the interconnections among the theoretical model variables as a function of sex (boys and girls).

To assess how well the proposed structural equation model (SEM) fits the observed data, several fit indices were employed. While the chi-square (χ^2^) test is traditionally used to evaluate model adequacy, with non-significant *p*-values suggesting a good fit, this statistic is known to be sensitive to sample size, as highlighted by Byrne [37]. Thus, a combination of complementary fit indices was also considered to ensure a more robust evaluation. These included the comparative fit index (CFI), incremental fit index (IFI), normalised fit index (NFI) and the Tucker–Lewis Index (TLI). A threshold of 0.90 or above was taken as indicative of acceptable fit, while values exceeding 0.95 were interpreted as evidence of excellent model fit. Additionally, the root mean square error of approximation (RMSEA) was calculated, with values below 0.08 indicating a reasonable fit and those under 0.05 reflecting a very good fit.

## 3. Results

Table 1 presents the descriptive characteristics pertaining to the study sample, according to sex. In this sense, boys reported lower English scores (6.60 ± 1.90 vs. 7.30 ± 2.05; *p* = 0.009; d = 0.354) and Language scores (6.33 ± 1.68 vs. 6.80 ± 1.77; *p* = 0.047; d = 0.272) than girls. In contrast, boys reported higher PA engagement than girls (2.87 ± 0.57 vs. 2.50 ± 0.67; *p* < 0.001; d = 0.594).

**Figure 2:**
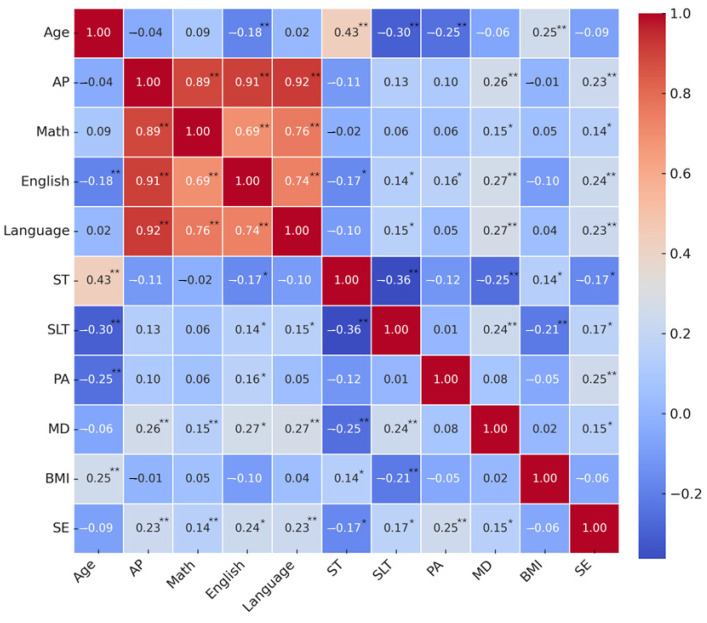
Colour map presenting the correlation coefficients produced between variables adjusted for sex. PA: physical activity engagement; ST: screen time; SLT: sleep time; MD: Mediterranean diet adherence; BMI: body mass index; SE: self-esteem; AP: academic performance; * *p* < 0.05, ** *p* < 0.01.

Figure 2 presents the correlation coefficients produced between study variables, whilst controlling for sex. Firstly, age was positively associated with ST (r = 0.433; *p* < 0.01) and with BMI (r = 0.251; *p* < 0.01) and inversely associated with English (r = −0.184; *p* < 0.01), SLT (r = −0.302; *p* < 0.01) and PA (r = −0.246; *p* < 0.01). Moreover, AP was positively associated with MD adherence (r = 0.255; *p* < 0.01) and SE (r = 0.228; *p* < 0.01). Specifically, Mathematics score was positively associated with MD adherence (r = 0.146; *p* < 0.05) and SE (r = 0.145; *p* < 0.05). English score was positively associated with SLT (r = 0.140; *p* < 0.05), PA (r = 0.163; *p* < 0.05), MD adherence (r = 0.265; *p* < 0.01) and SE (r = 0.237; *p* < 0.01), and inversely associated with ST (r = −0.166; *p* < 0.05). Also, Language score was positively associated with SLT (r = 0.151; *p* < 0.05), MD adherence (r = 0.272; *p* < 0.01), and with SE (r = 0.230; *p* < 0.01). Furthermore, ST was positively associated with BMI (r = 0.142; *p* < 0.05) and inversely associated with SLT (r = −0.363; *p* < 0.01), MD adherence (r = −0.252; *p* < 0.01) and SE (r = −0.169; *p* < 0.05). At the same time, SLT was positively correlated with MD adherence (r = 0.236; *p* < 0.01) and SE (r = 0.169; *p* < 0.05) and inversely associated with BMI (r = −0.214; *p* < 0.01). Finally, SE was positively associated with PA (r = 0.246; *p* < 0.01) and MD adherence (r = 0.148; *p* < 0.05).

**Figure 3:**
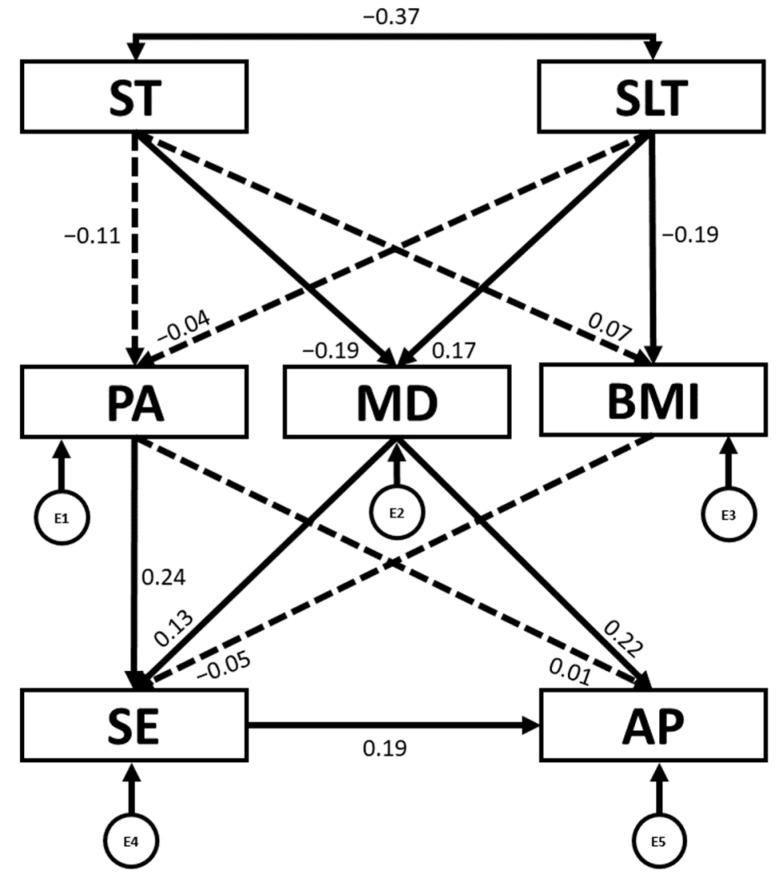
Structural equation model for the overall sample. PA: physical activity engagement; ST: screen time; SLT: sleep time; MD: Mediterranean diet; BMI: body mass index; SE: self-esteem; AP: academic performance. E1–E5: error terms. ←: directional relationships between variables; ↔: correlation between two variables. ← discontinuous: non-significant directional relationships between variables.

Table 2 and Figure 3 present the regression weights and standardized regression weights pertaining to the SEM constructed for the overall sample. The chi-square statistic was not significant (χ^2^ = 9.6; df = 8; *p* = 0.293), indicating that the null hypothesis of good model fit could not be rejected, as expected in structural equation modelling. This suggests that the proposed model fits the data well. Furthermore, other standardized fit indices were consulted that are less sensitive to sample size [37]. In this sense, IFI, TLI and CFI were all excellent, being 0.985, 0.954 and 0.985, respectively. Likewise, the NFI of 0.915 was adequate. At the same time, an excellent RMSEA value of 0.031 was produced.

The revised model explained 1% of total variance for PA, 8.6% for MD adherence, 4.9% for BMI, 7.7% for SE and 9.5% for AP.

Firstly, it can be observed that ST was negatively associated with MD (b = −0.189; *p* = 0.007) and SLT (b = −0.365; *p* < 0.001). In contrast, SLT was positively associated with MD (b = 0.166; *p* = 0.018) and negatively associated with BMI (b = −0.186; *p* = 0.009). Furthermore, PA (b = 0.237; *p* < 0.001) and MD (b = 0.130; *p* = 0.047) were both positively associated with SE. Moreover, MD (b = 0.222; *p* < 0.001) and SE (b = 0.187; *p* = 0.005) were positively associated with AP.

**Figure 4:**
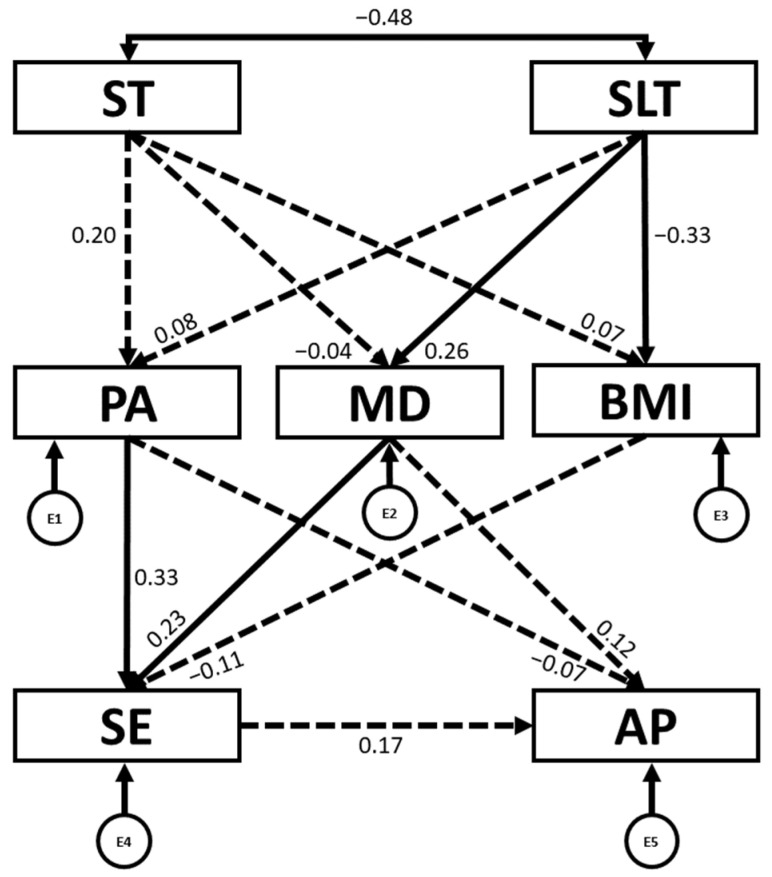
Structural equation model for boys. PA: physical activity engagement; ST: screen time; SLT: sleep time; MD: Mediterranean diet; BMI: body mass index; SE: self-esteem; AP: academic performance. E1–E5: error terms. ←: directional relationships between variables; ↔: correlation between two variables. ← discontinuous: non-significant directional relationships between variables.

Table 3 and Figure 4 present the regression weights and standardized regression weights pertaining to the SEM constructed for boys. The chi-square statistic was not significant (χ^2^ = 9; df = 8; *p* = 0.345), indicating that the null hypothesis of good model fit could not be rejected, as expected in structural equation modelling. This suggests that the proposed model fits the data well. Furthermore, other standardized fit indices were consulted that are less sensitive to sample size [37]. In this sense, IFI, TLI and CFI were all excellent, being 0.986, 0.956 and 0.983, respectively. Likewise, the NFI of 0.900 was adequate. At the same time, an excellent RMSEA value of 0.036 was produced.

The revised model explained 3.2% of total variance for PA, 7.7% for MD adherence, 13% for BMI, 18% for SE and 4.8% for AP.

Firstly, it can be observed that SLT was positively associated with MD (b = 0.256; *p* = 0.023) and negatively associated with BMI (b = −0.325; *p* = 0.003) and ST (b = −0.476; *p* < 0.001). Furthermore, PA (b = 0.332; *p* < 0.001) and MD (b = 0.235; *p* = 0.012) were positively associated with SE.

**Figure 5:**
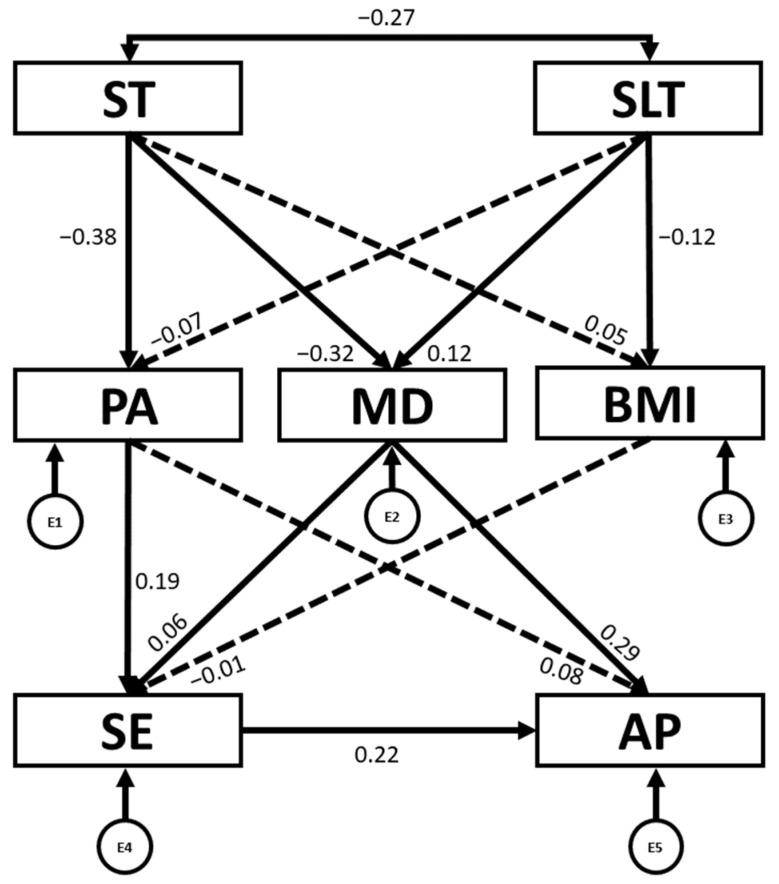
Structural equation model for girls. PA: physical activity engagement; ST: screen time; SLT: sleep time; MD: Mediterranean diet; BMI: body mass index; SE: self-esteem; AP: academic performance. E1–E5: error terms. ←: directional relationships between variables; ↔: correlation between two variables. ← discontinuous: non-significant directional relationships between variables.

Table 4 and Figure 5 present the regression weights and standardized regression weights pertaining to the SEM constructed for girls. The chi-square statistic was not significant (χ^2^ = 4.4; df = 8; *p* = 0.816), indicating that the null hypothesis of good model fit could not be rejected, as expected in structural equation modelling. This suggests that the proposed model fits the data well. Furthermore, other standardized fit indices were consulted that are less sensitive to sample size [37]. In this sense, IFI, TLI and CFI were all excellent, being 1.050, 1.161 and 1.000, respectively. Likewise, the NFI of 0.944 was adequate. At the same time, an excellent RMSEA value of <0.001 was produced.

The revised model explained 13.3% of total variance for PA, 13.4% for MD adherence, 2% for BMI, 4.3% for SE and 16.7% for AP.

Firstly, it can be observed that ST was negatively associated with PA (b = −0.378; *p* < 0.001), MD (b = −0.315; *p* < 0.001) and SLT (b = −0.271; *p* = 0.004). PA was positively associated with SE (b = 0.190; *p* = 0.034). Moreover, MD (b = 0.295; *p* < 0.001) and SE (b = 0.224; *p* = 0.008) were positively associated with AP.

## 4. Discussion

### 4.1. Modelling Lifestyle and Academic Outcomes in School-Aged Youth

The present research reveals important connections between ST, SLT, PA, MD adherence, BMI, SE and AP in high-SES school-aged youth from southern Spain. Other studies have sought to explain the same aforementioned associations as a function of SES [8]. However, when this effect is minimized, as was the case in the present sample, which was composed of high-SES participants who theoretically have greater access to resources, these relationships still persist. This suggests that such patterns are not solely driven by socioeconomic factors and warrant further investigation.

Firstly, a bidirectional negative association was observed between ST and SLT in the present study. In young people, the excessive use of electronic devices has been linked to delayed sleep onset and wake time [38], which may explain why increased ST correlates with shorter SLT. Furthermore, screen use during the period between going to bed and attempting to fall asleep has also been associated with reduced total SLT [38]. Activities such as watching videos or streaming series in bed tend to prolong engagement with content, driven by recommendation algorithms and auto-play functions [39,40], which can delay the onset of sleep. These disruptions in the sleep–wake cycle among school-aged youth have been associated with a higher risk of developing sleep disorders [41].

ST has also been negatively associated with MD adherence. According to Bibiloni et al. [42], Spanish schoolchildren who complied with ST recommendations on both weekdays and weekends tended to exhibit higher MD adherence. Conversely, exceeding the recommended ST has been linked to increased consumption of ultra-processed foods such as sweets, snacks and sugary beverages [20], which may partially explain this association. Notably, among Spanish school-aged youth, mobile phone use has shown a stronger association with low MD adherence, increased sedentary behaviour, higher intake of sugary drinks, unhealthy eating patterns and shorter SLT compared to other screen-related habits such as television viewing [43].

In the present study, a positive relationship between SLT and MD adherence was also observed. In this sense, sleep deprivation is a known risk factor for obesity in school-aged youth [44]. Córdova et al. [45] reported that youth who slept less had a 51% higher risk of adopting unhealthy dietary habits. This relationship may be mediated by hormonal disruptions affecting appetite regulation due to insufficient sleep, as well as by increased time spent awake, which elevates the likelihood of food consumption and exposure to food-related stimuli, such as advertisements on screens [46].

At the next level of the SEM, healthy habits such as PA and MD adherence have been positively associated with SE. In school-aged youth from southern Spain, a positive relationship has been found between these health behaviours and SE [47]. Additionally, other studies suggest that functional PA, as opposed to exercise driven by body image concerns, is more strongly associated with positive SE in youth [48]. This may be due to the fact that functional health behaviours are related to more positive body image perception [49], which in turn enhances young people’s overall emotional well-being.

In recent decades, the relationship between PA and AP has been widely studied in school-aged youth, yielding positive and promising results [1]. Although the present study did not find a significant direct relationship between PA and AP, this absence may be attributed to the self-reported nature of the PA assessment and the use of academic grades as a general indicator of cognitive performance. Previous studies using objective measures, such as accelerometery, to quantify exercise intensity and neurocognitive tests have reported significant positive associations between physical exercise, cognition and AP across different age groups [50]. However, a systematic review of randomized controlled trials examining the effects of PA on cognition concludes that further empirical evidence is needed in this area. The authors report that the observed effects tend to be small when controlling for key moderators and may become statistically insignificant after adjusting for publication bias [51]. In this regard, findings of the present study should be interpreted with caution, in consideration of its methodological limitations and the need for future research that employs objective measures and more rigorous experimental designs.

Furthermore, MD adherence was positively associated with AP in the present study. Other studies have observed MD adherence to be associated with multiple cognitive functions, including memory, attention, creativity, language skills, and executive functions [52]. Among older Spanish students, some specific dietary components have been found to be positively associated with AP including olive oil, vegetables, fruits, legumes, and fish, whereas high consumption of sweets and carbonated beverages have exhibited negative associations [16]. Thus, MD adherence may be considered a relevant factor in the analysis of the relationship between healthy lifestyle habits, cognition, and AP, due to the potential effect of the molecular basis of certain foods on cognition [53].

Finally, the present study found a positive relationship between SE and AP. Existing literature suggests that SE is a relevant precursor that can enhance school engagement and motivation towards AP [54]. This association may be explained by the fact that students with high SE tend to be less susceptible to the negative effects of failure in academic tests [55], which may, in turn, promote persistence in the face of academic challenges and the use of more effective learning strategies.

An examination of the positive effects of healthy habits on AP cannot, therefore, be undertaken without consideration of the psychological factors that may influence these relationships.

### 4.2. Model Associations as a Function of Sex

Separate SEMs were also constructed as a function of sex. Both models demonstrated excellent fit, confirming the structural validity of the proposed relationships. However, the comparison between models for boys and girls revealed relevant differences in both the explained variance and the direction and strength of relationships between variables.

The model for boys explained a greater proportion of variance in psychological variables, such as SE (18%) and BMI (13%), compared to the model for girls (4.3% and 2%, respectively). Conversely, the model for girls explained a higher proportion of the variance in PA (13.3%) and AP (16.7%) than the model for boys (3.2% and 4.8%, respectively).

Firstly, ST emerged as a stronger determinant in the model for girls, exhibiting negative associations with PA (b = −0.378; *p* < 0.001) and MD adherence (b = −0.315; *p* < 0.001), a pattern not observed in boys (see Figure 4 and Figure 5). This may be explained by the tendency of boys to use ST for more active purposes, such as playing video games or sports-related content, while girls are more likely to engage with social media in order to access content related to music, fashion, health, beauty, and celebrities [56]. In this sense, girls are particularly influenced by fitness and body-image content on social media, which may affect their eating habits and body image perceptions [57], often leading to dysfunctional exercise behaviours or eating with the intent to change their body shape and control weight rather than for health-related reasons.

In both sexes, a positive association was found between PA and SE, although this relationship was stronger in boys (b = 0.332; *p* < 0.001) than in girls (b = 0.190; *p* = 0.034) (see Figure 4 and Figure 5). As observed in the present study and supported by previous research, boys tend to engage more frequently in PA than girls, both within and outside the school setting, and show greater motivation towards PA [58]. This suggests that boys may derive SE more directly from physical competence, AP, and other achievement-related factors, which leads PA to have a stronger impact on their self-worth. In contrast, body image perception appears to be a more central factor in the case of girls [59] when it comes to influencing both their PA engagement and development of their SE.

Moreover, in the girls’ model, MD adherence (b = 0.295; *p* < 0.001) and SE (b = 0.224; *p* = 0.008) were positively associated with AP, a pattern not observed in the boys’ model (see Figure 4 and Figure 5). Previous studies suggest that girls may benefit more from higher SE in academic contexts, as boys have been found to show lower academic self-confidence in comparison [60]. Additionally, girls tend to report greater MD adherence, possibly due to increased concern about food naturalness, weight, and body shape [61]. In this case, this may actually have a positive influence on their AP, as this dietary pattern has been associated with better AP [5].

From a theoretical perspective, gendered socialisation processes might explain these differences. According to social cognitive theory of gender development [62], boys and girls internalise distinct expectations regarding AP and self-regulation. Girls often experience stronger social pressure to perform well academically and maintain healthy behaviours, as predicted by expectancy-value theory [63] and the concept of parental and societal academic expectations [64], which may reinforce the associations among SE, MD adherence and AP. Conversely, boys tend to base their self-concept on other domains such as physical competence or social status, in line with self-concept theory [65] and the gender similarities/differences framework [66], making these associations less pronounced. In terms of sex differences, it is suggested that research on the influence of healthy habits on AP should not be limited to directional studies (e.g., PA and cognition). Instead, it is essential to account for additional factors that may influence this relationship, such as MD adherence or SE, which have been shown to play a relevant role in this context.

### 4.3. Practical Implications and Future Study Perspectives

Present findings provide relevant implications for the design of scientific, school-based, family, and community interventions aimed at promoting a healthy lifestyle and improving AP in school-aged youth.

First, the need to reduce ST in this population becomes evident, with the importance of mobile device being made clear by existing literature on the topic. In this regard, future interventions should focus on encouraging appropriate and mindful use of digital devices, placing particular emphasis on monitoring ST and the type of content consumed.

Schools and families could benefit from awareness campaigns on the responsible use of electronic devices, in line with WHO guidelines [7]. These interventions should include strategies to establish healthy digital routines, such as setting consistent ST limits, particularly in the hours before bedtime, in order to avoid sleep delay. Moreover, alternative activities like reading or active play should be promoted. In this context, the interest of this population in digital technologies could be leveraged by incorporating active video games as an opportunity to transform sedentary ST into active time, while also providing potential cognitive benefits [67]. Additionally, such alternative activities may also reduce the tendency to consume unhealthy foods during screen exposure, thereby contributing to healthier dietary patterns.

While the growing interest of young people in digital technologies can be leveraged to promote health, it is important not to overlook the need for directly reducing screen exposure through the inclusion of alternative activities. Programs should, therefore, incorporate outdoor, cooperative and socially engaging PA that encourages movement, teamwork and real-world interaction. These experiences not only help to counteract the sedentary and isolating nature of excessive screen use but also foster essential social and emotional skills. Thus, combining technological engagement with opportunities for physical and social connection represents a more balanced and effective approach to improving both health and academic outcomes.

Furthermore, the continued implementation of curriculum-based interventions aimed at promoting healthy habits remains essential, as these have shown positive effects on both physical health and the emotional and mental well-being of youth [68]. However, the findings of the present study raise questions about whether the dissemination of information and promotion of PA or nutrition alone is sufficient, considering the persistently high rates of physical inactivity and low adherence to a healthy diet found among this population, despite previous efforts. Thus, based on present findings, it is suggested that comprehensive interventions be developed that not only promote and facilitate healthy lifestyle practices but also address underlying mental health issues, such as low SE, that may act as barriers and are often overlooked.

Regarding sex-based differences, interventions should also be tailored to the specific needs and characteristics of boys and girls. For instance, in the case of girls, the findings suggest a need to address the critical use of social media in order to counteract the influence of content that promotes distorted body image and unhealthy behaviours (e.g., dysfunctional PA and disordered eating) in pursuit of the unrealistic beauty standards that are often portrayed on such media.

Finally, it is worth highlighting that the present study also found that, as age increases, ST tends to rise, while PA and AP decline. It is, therefore, essential for both the scientific and educational communities to prioritize early and preventive intervention strategies from an early age.

Overall, schools could implement screen-free periods, movement breaks and after-school clubs that promote outdoor and cooperative activities. Families should be encouraged to adopt device-free dinners, introduce reward systems for balanced screen and PA time, and engage in shared active routines. Communities could offer accessible programs that combine physical, social and digital activities, as well as mentoring or counselling services to support mental well-being. Such coordinated strategies help reduce sedentary behaviour, encourage healthy lifestyles, and foster social and emotional skills from an early age.

### 4.4. Study Limitations and Future Suggestions

The present study presents a number of limitations that should be taken into consideration, which, in turn, open avenues for improvement in future research aimed at enhancing the reliability and scope of the findings.

Firstly, data was gathered through self-reported questionnaires, which may entail a certain degree of measurement error due to potential biases such as recall bias or social desirability bias. However, the instruments employed (PAQ-C, KIDMED, and the Rosenberg Self-Esteem Scale) have demonstrated sufficient validity and reliability for use in this specific population and so their likely impact on the final results is considered minimal. Nevertheless, future studies could incorporate affordable and practical technological tools, such as accelerometers or heart rate monitors, which would allow for the collection of more objective and accurate data on the frequency and intensity of PA over specific time intervals.

Secondly, a cross-sectional design was used, with a single data collection point in a specific sample at a given moment. While this methodology allows for the identification of associations between variables, it does not enable the establishment of causal relationships. Longitudinal studies or experimental designs are recommended to more robustly explore the causal links between examined variables.

In addition, the sample was drawn from a single mixed-funding school located in the city of Granada. This characteristic limits the generalizability of findings to other geographic areas or educational settings. Thus, it would be beneficial to replicate the study in diverse regions and types of schools in order to examine the consistency of the findings across different contexts and populations.

Finally, the proportion of variance explained by the models, particularly for AP, was modest. This indicates that other relevant predictors (such as family environment, motivation and school climate) were not captured in the current model. Moreover, although SEM was selected for its capacity to test complex interrelations among multiple variables, the sex-specific subsamples available in the present study were relatively small, which may have affected the stability of parameter estimates. These results should, therefore, be interpreted with caution.

## 5. Conclusions

The present study successfully developed an explanatory model of the relationships between lifestyle habits, psychological variables, and AP in school-aged youth, revealing consistent patterns regardless of SES. The findings support the existence of an association between healthy habits and SE in AP. Additionally, significant sex differences were identified, which highlights the need to tailor health promotion and AP strategies to the specific characteristics of each group. These results underscore the importance of implementing educational, family, and community programs from an early age that are aimed at reducing sedentary behaviour, promoting the reasonable use of technology, and encouraging the adoption of healthy habits. Future research employing longitudinal or experimental methodologies, as well as objective measures of PA, will help to confirm and generalise these findings across diverse school settings.

## Figures and Tables

**Table 1:** Sample characteristics according to sex.

	Sex (*n* = 217)		*p* Value	Cohen’s d	Total
	Boys (*n* = 95)	Girls (*n* = 122)			
Age	13.96 ± 1.30	13.88 ± 1.32	0.470	0.061	13.88 ± 1.32
AP	6.60 ± 1.51	7.03 ± 1.70	0.067	0.267	6.84 ± 1.63
Math	6.87 ± 1.46	7.01 ± 1.77	0.724	0.086	6.95 ± 1.64
English	6.60 ± 1.90	7.30 ± 2.05	0.009	0.354	6.99 ± 2.01
Language	6.33 ± 1.68	6.80 ± 1.77	0.047	0.272	6.59 ± 1.74
ST	3.67 ± 2.17	3.36 ± 1.73	0.509	0.158	3.50 ± 1.94
SLT	8.54 ± 0.90	8.63 ± 0.99	0.723	0.095	8.59 ± 0.95
PA	2.87 ± 0.57	2.50 ± 0.67	<0.001	0.594	2.66 ± 0.65
MD	6.04 ± 2.46	5.93 ± 2.25	0.766	0.047	5.98 ± 2.34
BMI	19.37 ± 3.04	19.62 ± 3.67	0.746	0.074	19.51 ± 3.40
SE	28.36 ± 3.68	27.98 ± 3.94	0.452	0.100	28.14 ± 3.82

Note: PA: physical activity engagement; ST: screen time; SLT: sleep time; MD: Mediterranean diet adherence; BMI: body mass index; SE: self-esteem; AP: academic performance.

**Table 2:** Regression weights for the overall sample.

Association Between Variables			RW				SRW
			Estimation	SE ^2^	CR	*p* Value	Estimation
PA	←	ST	−0.036	0.025	−1.464	0.143	−0.106
PA	←	SLT	−0.028	0.050	−0.549	0.583	−0.040
MD	←	ST	−0.228	0.084	−2.701	0.007	−0.189
MD	←	SLT	0.408	0.172	2.369	0.018	0.166
BMI	←	ST	0.125	0.125	0.998	0.319	0.071
BMI	←	SLT	−0.668	0.256	−2.611	0.009	−0.186
SE ^1^	←	PA	1.381	0.381	3.624	<0.001	0.237
SE ^1^	←	MD	0.212	0.107	1.986	0.047	0.130
SE ^1^	←	BMI	−0.053	0.073	−0.726	0.468	−0.048
AP	←	PA	−0.012	0.166	−0.074	0.941	−0.005
AP	←	MD	0.155	0.045	3.401	<0.001	0.222
AP	←	SE ^1^	0.080	0.029	2.780	0.005	0.187
SLT	↔	ST	−0.667	0.132	−5.040	<0.001	−0.365

Note. PA: physical activity engagement; ST: screen time; SLT: sleep time; MD: Mediterranean diet; BMI: body mass index; SE ^1^ self-esteem; AP: academic performance; RW: regression weight; SRW: standardized regression weight; SE ^2^: standard error; CR: critical ratio; ←: directional relationships between variables; ↔: correlation between two variables. Bold *p*-values indicate statistical significance (<0.05).

**Table 3:** Regression weights for boys.

Association Between Variables			RW				SRW
			Estimation	SE ^2^	CR	*p* Value	Estimation
PA	←	ST	0.053	0.030	1.751	0.080	0.202
PA	←	SLT	0.050	0.073	0.683	0.495	0.079
MD	←	ST	−0.047	0.128	−0.365	0.715	−0.041
MD	←	SLT	0.696	0.307	2.269	0.023	0.256
BMI	←	ST	0.090	0.154	0.588	0.557	0.064
BMI	←	SLT	−1.098	0.369	−2.975	0.003	−0.325
SE ^1^	←	PA	2.148	0.605	3.550	<0.001	0.332
SE ^1^	←	MD	0.353	0.141	2.501	0.012	0.235
SE ^1^	←	BMI	−0.135	0.114	−1.187	0.235	−0.111
AP	←	PA	−0.185	0.282	−0.657	0.511	−0.070
AP	←	MD	0.073	0.064	1.136	0.256	0.118
AP	←	SE ^1^	0.067	0.045	1.498	0.134	0.165
SLT	↔	ST	−0.992	0.221	−4.168	<0.001	−0.476

Note. PA: physical activity engagement; ST: screen time; SLT: sleep time; MD: Mediterranean diet; BMI: body mass index; SE ^1^: self-esteem; AP: academic performance; RW: regression weight; SRW: standardized regression weight; SE ^2^: standard error; CR: critical ratio; ←: directional relationships between variables; ↔: correlation between two variables. Bold *p*-values indicate statistical significance (<0.05).

**Table 4:** Regression weights for girls.

Association Between Variables			RW				SRW
			Estimation	SE ^2^	CR	*p* Value	Estimation
PA	←	ST	−0.147	0.034	−4.304	<0.001	−0.378
PA	←	SLT	−0.051	0.060	−0.849	0.396	−0.075
MD	←	ST	−0.410	0.114	−3.582	<0.001	−0.315
MD	←	SLT	0.277	0.201	1.378	0.168	0.121
BMI	←	ST	0.115	0.198	0.582	0.561	0.054
BMI	←	SLT	−0.430	0.348	−1.234	0.217	−0.115
SE ^1^	←	PA	1.115	0.525	2.123	0.034	0.190
SE ^1^	←	MD	0.107	0.157	0.683	0.495	0.061
SE ^1^	←	BMI	−0.014	0.095	−0.142	0.887	−0.013
AP	←	PA	0.199	0.215	0.928	0.354	0.079
AP	←	MD	0.222	0.063	3.523	<0.001	0.295
AP	←	SE ^1^	0.096	0.036	2.637	0.008	0.224
SLT	↔	ST	−0.458	0.159	−2.878	0.004	−0.271

Note. PA: physical activity engagement; ST: screen time; SLT: sleep time; MD: Mediterranean diet; BMI: body mass index; SE ^1^: self-esteem; AP: academic performance; RW: regression weight; SRW: standardized regression weight; SE ^2^: standard error; CR: critical ratio; ←: directional relationships between variables; ↔: correlation between two variables. Bold *p*-values indicate statistical significance (<0.05).

## Data Availability

The data presented in this study are available on request from the corresponding author. The data are not publicly available due to the database not being shared in any public repository.

## Abbreviations

The following abbreviations are used in this manuscript:

ST	Screen Time
SLT	Sleep Time
PA	Physical Activity Engagement
MD	Mediterranean diet
BMI	Body Mass Index
SE	Self-Esteem
AP	Academic Performance
SES	Socioeconomic Status
